# Supplementary figures and images for: Structures of the human cholecystokinin 1 (CCK1) receptor bound to Gs and Gq mimetic proteins provide insight into mechanisms of G protein selectivity
Source: PLoS Biol. 2021 Jun 4;19(6):e3001295. doi: 10.1371/journal.pbio.3001295 (PMC8208569; doi:10.1371/journal.pbio.3001295)

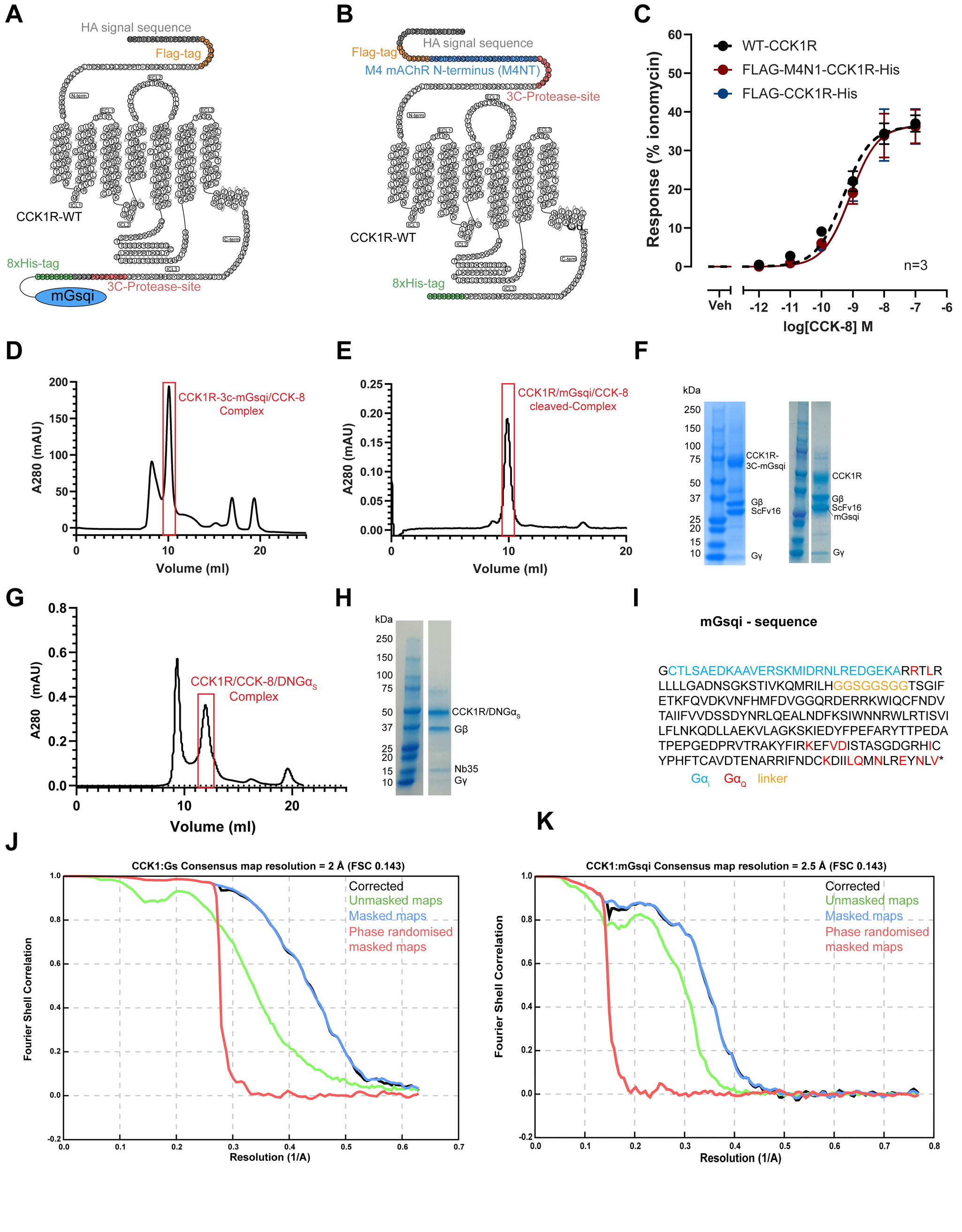

Supplement: S1 Fig — (A, B) Snake plot of the CCK1R expression constructs for formation of mGsqi (A) or Gs (B) complexes. The construct for mGsqi complex formation contained an N-terminal HA signal sequence (grey shading), followed by a FLAG epitope tag (yellow shading), with a 3C protease cleavage site (red shading) inserted at the carboxyl terminus followed by an 8-His tag (green shading) and the mGsqi (blue schematic). For Gs complex formation, the construct contained an N-terminal HA signal sequence, followed by a FLAG epitope tag, a M4 mAChR N-terminal sequence (blue shading), and a 3C-cleavage site (red sequence) with an 8-His tag fused to the carboxyl terminus. (C) The expression constructs (red circles or blue circles) were cloned into a mammalian expression vector and concentration-responses to CCK-8 in an iCa2+ mobilisation assay were established relative to WT (black circles). (D, E) SEC of affinity purified CCK-8/CCK1R/mGαsqi/Gβ1γ2/scFv16 complex before (D) and after (E) sample cleavage with 3C protease; the peak used for SDS-PAGE analysis is boxed in red. (F) Coomassie blue stained SDS-PAGE of the peak sample from (D) (left panel) or (E) (right panel). (G) SEC of affinity purified CCK-8/CCK1R/DNGαs/Gβ1γ2/Nb35 complex. (H) Coomassie blue stained SDS-PAGE of the peak sample from (G). (I) amino acid sequence of the mGsqi chimera illustrating the origin of the different segments. (J, K) Gold standard FSC curves for the final map and map validation from half maps showing the overall nominal resolutions of 2.5 Å for the CCK1R-“Gq mimetic” complex (J) and 2.0 Å for the CCK1R–Gs complex (K). The data used to generate graph S1C, and the SEC traces in S1D, S1E, and SG are available in S1 Data. Uncropped gels for (F) and (H) are provided as S2 Data. Data for calculation of the FSC curves in S1J and S1K can be accessed in EMDB-23749 and EMDB-23750. CCK, cholecystokinin; CCK1R, cholecystokinin type 1 receptor; FSC, Fourier shell correlation; HA, hemagglutinin; SEC, size exclusion chroma [file pbio.3001295.s001.tif]

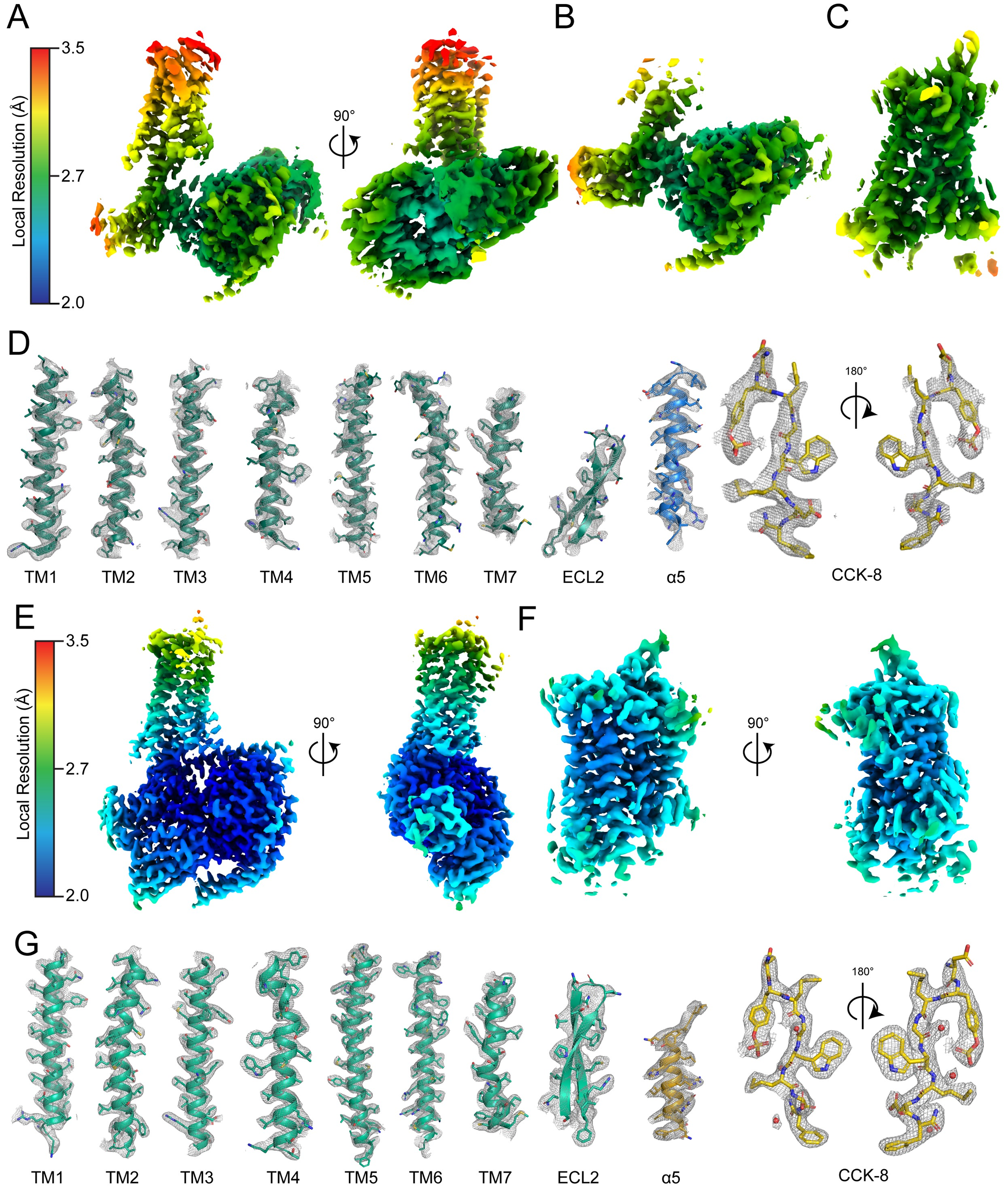

Supplement: S2 Fig — Local resolution of (A) the consensus map, (B) G protein–focused refinement, and (C) receptor-focused refinement of the CCK-8/CCK1R/mGαsqi/Gβ1γ2/scFv16 complex. (D) Density maps and models are illustrated for all 7 transmembrane helices and ECL2 of CCK1R, the αH5 of the Gα subunit, and the CCK-8 peptide. (E, F) Local resolution of the consensus (E) and receptor-focused (F) maps for the CCK-8/CCK1R/DNGαs/Gβ1γ2/Nb35 complex. (G) Density maps and models are illustrated for all 7 transmembrane helices and ECL2 of CCK1R, the αH5 of the Gα subunit, and the CCK-8 peptide. Protein backbone is displayed in ribbon format with amino acid side chains in stick representation, coloured by heteroatom. The cryo-EM density was zoned at 1.8 Å. αH5, α5 helix; CCK, cholecystokinin; CCK1R, cholecystokinin type 1 receptor; cryo-EM, cryo-electron microscopy. (TIF) [file pbio.3001295.s002.tif]

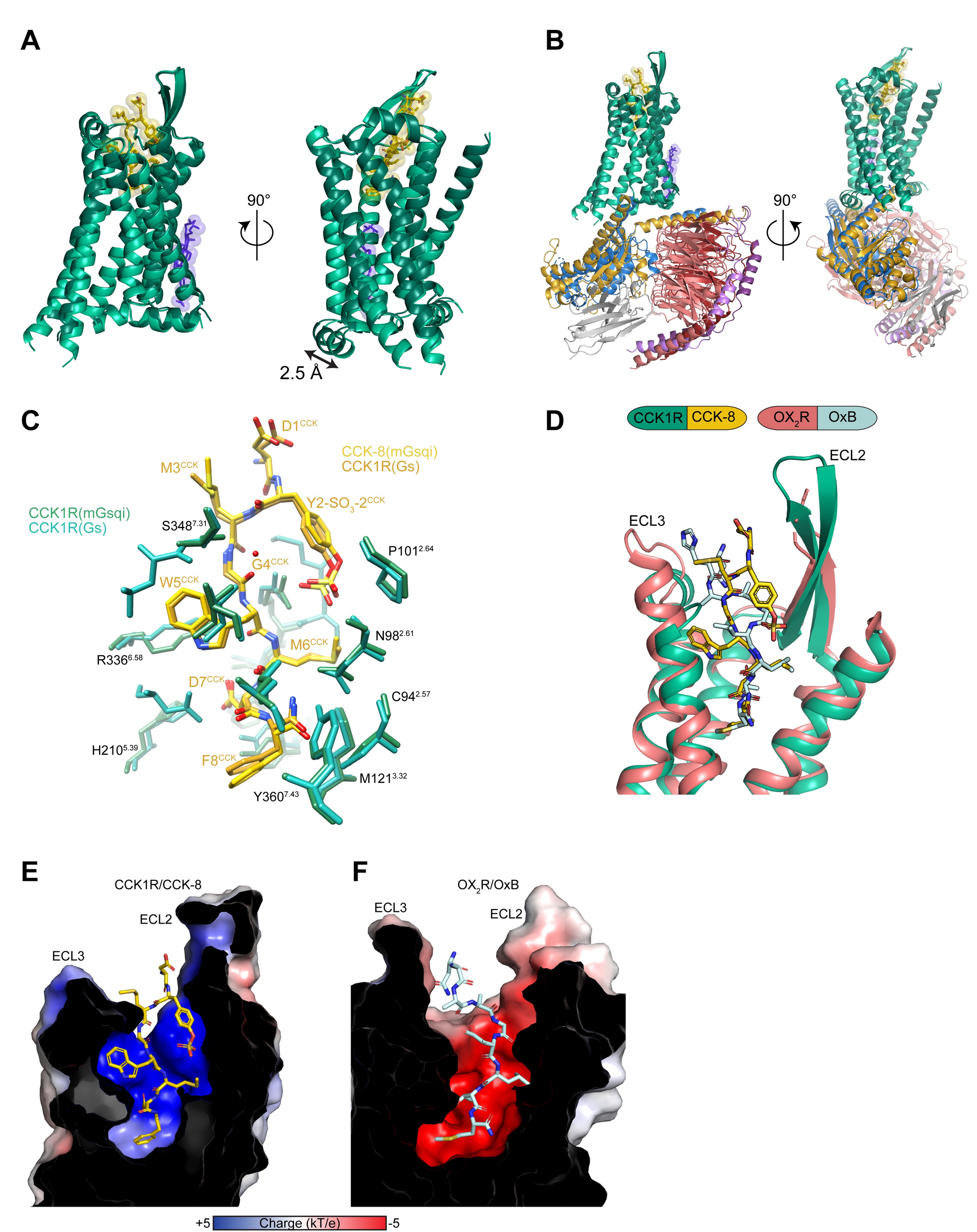

Supplement: S3 Fig — (A, B) Alignment of the 2 structures. (A) Alignment of receptor and CCK-8 peptide. The largest difference in the CCK1R was in the location of ICL2 that is further away from the receptor core in the complex with the Gq mimetic protein. (B) Alignment of the full complex illustrating differences in the engagement and orientation of the G proteins. The receptor and G proteins are displayed in ribbon format. (C) Overlay of CCK-8 and interacting CCK1R residues in the binding pocket for the Gs and Gq mimetic complexes. The CCK-8 peptide and modelled cholesterol are displayed in ball and stick representation. The CCK-8 residues are displayed in stick format, coloured by heteroatom (Gs complex, gold; Gq mimetic complex, yellow). CCK1R side chains that interact with the peptide are shown in stick format (Gs complex, light green; Gq mimetic complex, dark green). (D) Comparison of the CCK-8/CCK1R with OxB/OX2R (blue ligand, pink receptor) binding pockets. The peptides overlap in the location of the amidated carboxyl-terminal tetrapeptide but differ in conformation and position of N-terminal amino acids. CCK-8 interacts more with ECL2 than ECL3, while the reverse is true for OxB. (E) Surface electrostatic potential of the CCK1R binding pocket. CCK1R has a very positive surface charge. (F) In contrast, the OX2R binding pocket has a very negative surface potential. CCK, cholecystokinin; CCK1R, cholecystokinin type 1 receptor. (TIF) [file pbio.3001295.s003.tif]

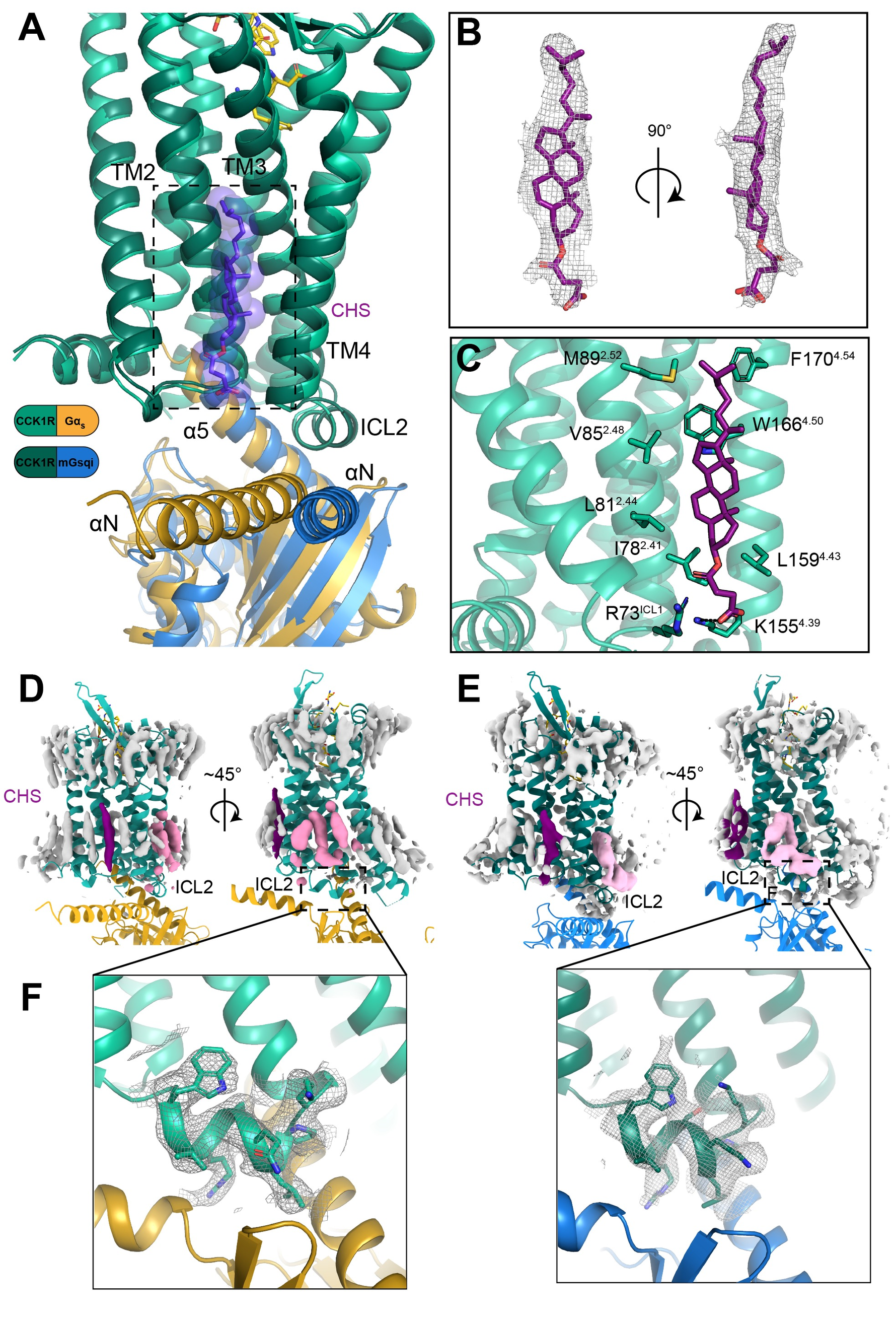

Supplement: S4 Fig — (A) Alignment of the Gs and mGsqi structures illustrating the location of the Gαs protein and modelled lipid. (B) Model of the cholesteryl hemi succinate (CHS) in the cryo-EM density. (C) The modelled CHS interacts with TM2 and TM4 of CCK1R. (D, E) Receptor-focused maps for the Gs protein-complex (D) and Gq mimetic protein complex (E) are shown, coloured by component (CCK1R, green; CCK-8, yellow; Gαs protein, gold; Gαq-mimetic protein, blue; unmodelled lipids, grey; putative CHS, purple). There is weak cryo-EM density in the predicted binding site for the allosteric cholesterol abutting ICL2 (pink density). (F) The cryo-EM density of ICL2 (zoned at 1.8 Å) in the Gs complex supports a single predominant conformation, while the density in the Gq mimetic complex is less well resolved. CCK, cholecystokinin; CCK1R, cholecystokinin type 1 receptor; CHS, cholesteryl hemisuccinate; cryo-EM, cryo-electron microscopy. (TIF) [file pbio.3001295.s004.tif]

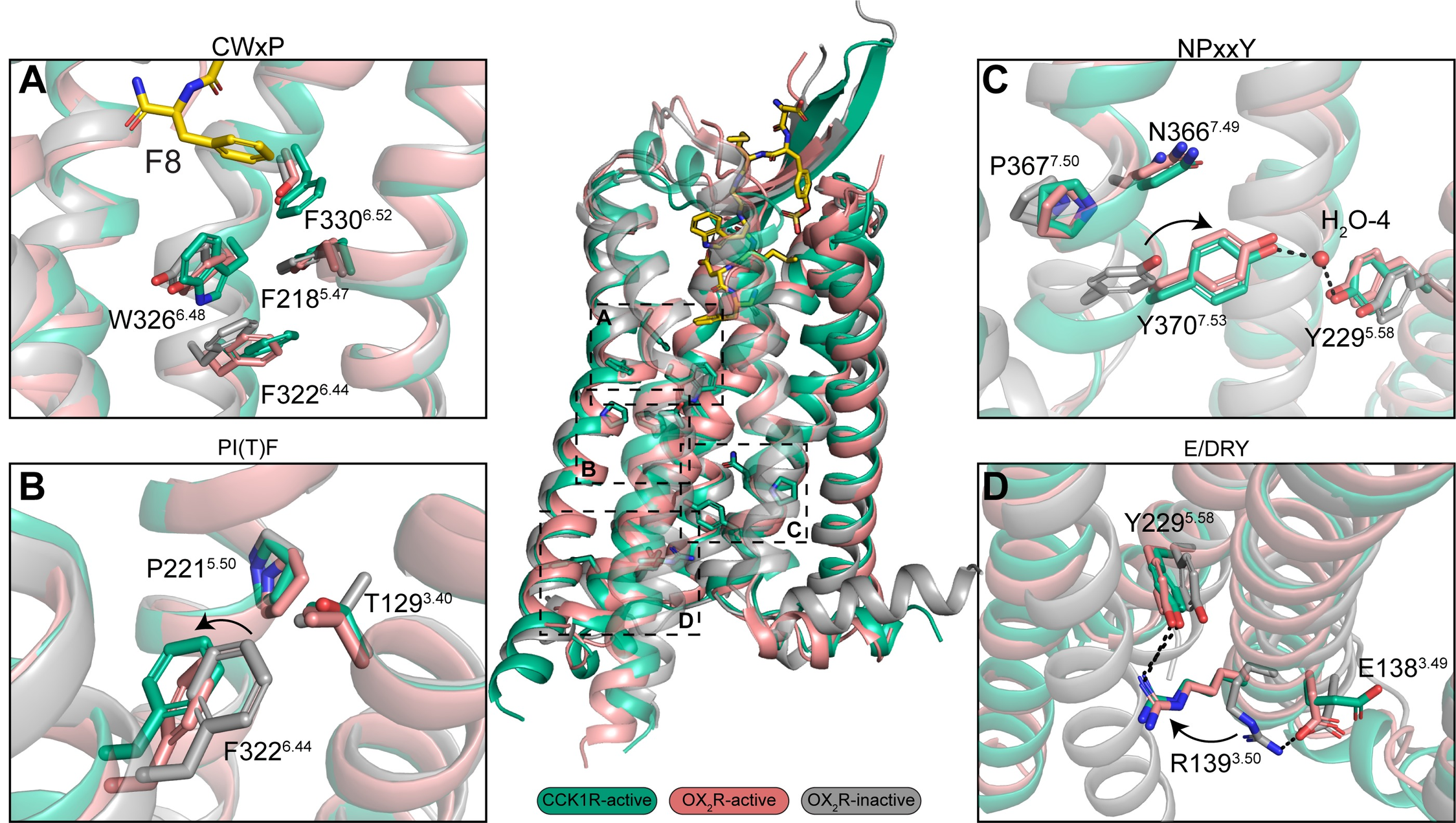

Supplement: S5 Fig — (A) CWxP motif. While there is no direct interaction between CCK-8 and W3266.48 of the CWxP motif, residue F8CCK interacts with F3306.52, which, in turn, interacts with W3266.48. Of note, residue 6.48 is not conserved in OX2R, and there is no movement between the inactive and active states at this position. Given the absence of an inactive CCK1R structure the role of the CWxP motif in CCK1R is unclear. Nevertheless, the position of W3266.48 stabilises the outward rotation of F3226.44 within the PI(T)F motif. (B) Comparison of the PI(T)F motif shows this activation motif is largely conserved between CCK1R and OX2R. Upon activation, there is a modest movement of F6.44 for OX2R. CCK1R is even further shifted away from T1293.40 and P2215.50. (C) NPxxY motif. Upon activation, there is a clear rotation of Y7.53 for OX2R. CCK1R Y3707.53 overlays with the active form of OX2R and forms a water mediated interaction Y2295.58. (D) E/DRY motif. In the OX2R, inactive state R3.50 forms a salt-bridge with E3.49; these residues are conserved in CCK1R. Upon activation, R3.50 rotates up and inwards to form a hydrogen bond with Y5.58, the position of these residues overlay with the active CCK1R. CCK, cholecystokinin; CCK1R, cholecystokinin type 1 receptor. (TIF) [file pbio.3001295.s005.tif]

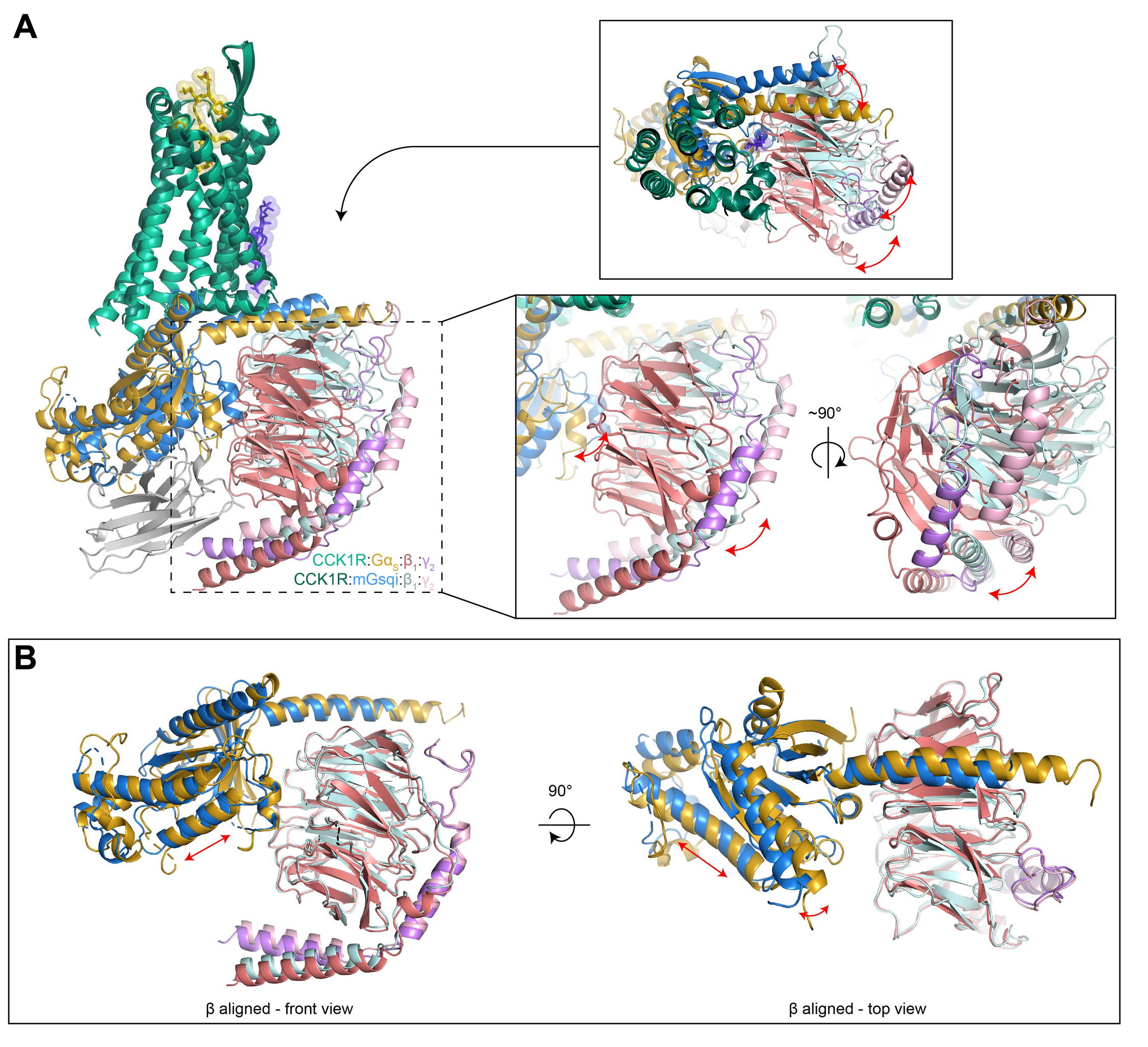

Supplement: S6 Fig — (A) Orientation of the G proteins following alignment on CCK1R. The protein backbone is displayed in ribbon format coloured according to the displayed legend. The CCK-8 peptide (yellow; stick and surface representation) and modelled CHS (purple, stick and surface representation) are also shown (main panel). Gs and mGsqi have a different angle of engagement, and this is propagated to larger changes in the relative positions of the Gβ and Gγ subunits (inset panels). (B) Alignment of the Gβ subunits reveals that the Gα subunits have distinct rotational and translational positions in the heterotrimer. CCK, cholecystokinin; CCK1R, cholecystokinin type 1 receptor; CHS, cholesteryl hemisuccinate. (TIF) [file pbio.3001295.s006.tif]

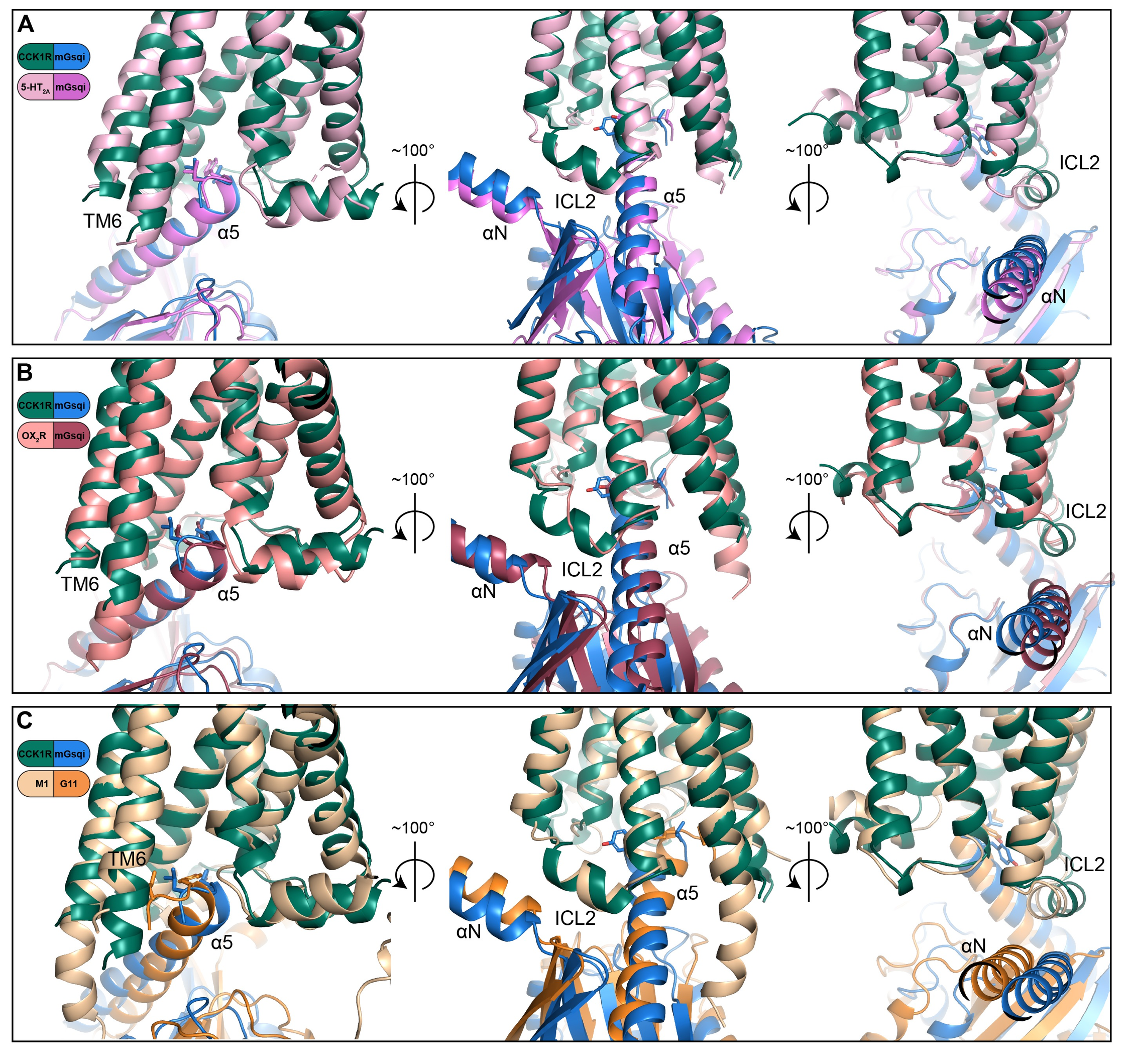

Supplement: S7 Fig — Each of the available Gq (Gq mimetic chimera) bound structures is displayed relative to the structure of the CCK1R–mGsqi complex. (A) CCK1R–mGsqi and 5HT2AR-mGsqi (PDB:6WHA). (B) CCK1R–mGsqi and OX2R-mGsqi (PDB:7L1U). (C) CCK1R–mGsqi and M1 mAChR-G11/i chimera [PDB:6OIJ). The structures are aligned to the CCK1R. The protein backbone is shown in ribbon format coloured according to the displayed legends. The carboxyl-terminal residues of the G protein αH5 are shown in stick representation coloured by heteroatom. All receptors have a similar, narrow, intracellular G protein binding cavity with only small differences in the angle or translational position of the αH5 when bound to the receptor (A–C), with greatest difference seen between CCK1R and M1 mAChR complexes (C). αH5, α5 helix; CCK, cholecystokinin; CCK1R, cholecystokinin type 1 receptor; GPCR, G protein–coupled receptor. (TIF) [file pbio.3001295.s007.tif]
