# Supplementary material for: Structures of the human cholecystokinin 1 (CCK1) receptor bound to Gs and Gq mimetic proteins provide insight into mechanisms of G protein selectivity
Source: PLoS Biol. 2021 Jun 4;19(6):e3001295. doi: 10.1371/journal.pbio.3001295 (PMC8208569; doi:10.1371/journal.pbio.3001295)
Supplement: S1 Table — (PDF) [file pbio.3001295.s008.pdf]

S1 Table: Data collection and refinement statistics.

Data Collection	CCK1R/Gs/CCK-8	CCK1R/mGsqi/CCK-8
Micrographs	7146	7182
Electron dose (e^-/A^2)	70.56	63.9
Voltage (kV)	300	300
Pixel size (Å)	0.65	0.65
Defocus range (μm)	0.5-1.5	0.5-1.5
Symmetry imposed	C1	C1
Particles (final map)	643k	444k
Resolution (0.143 FSC) (Å)	1.95	2.44
**Refinement**		
CC_{map_model}	0.72	0.68
Map sharpening B factor (Å^2)	-38.0	-73.2
**Model Quality**		
R.m.s. deviations		
Bond length (Å)	0.004	0.005
Bond angles (^{o})	0.782	0.914
Ramachandran		
Favoured (%)	98.47	98.27
Outliers (%)	0.00	0.00
Rotamer outliers (%)	0.45	0.0
C-Beta deviations (%)	0	0
Clashscore	2.22	4.48
MolProbity score	1.00	1.25
