# Supplementary material for: Structures of the human cholecystokinin 1 (CCK1) receptor bound to Gs and Gq mimetic proteins provide insight into mechanisms of G protein selectivity
Source: PLoS Biol. 2021 Jun 4;19(6):e3001295. doi: 10.1371/journal.pbio.3001295 (PMC8208569; doi:10.1371/journal.pbio.3001295)
Supplement: S2 Data — (PDF) [file pbio.3001295.s018.pdf]

Supp Figure 1F_LEFT

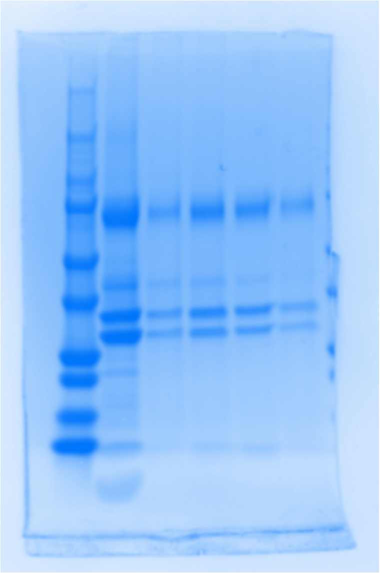

M = Bio-rad precission plus dual protein standards
L = Sample loaded onto SEC
# = Volume sample was collected from SEC (ml)

Supp Figure 1F_RIGHT

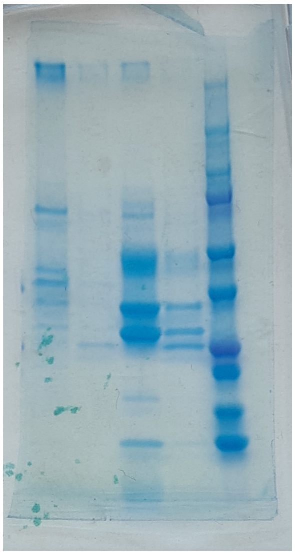

M = Bio-rad precission plus dual protein standards
C = Concentrated sample after SEC
# = Volume sample was collected from SEC (ml)

Supp Figure 1H

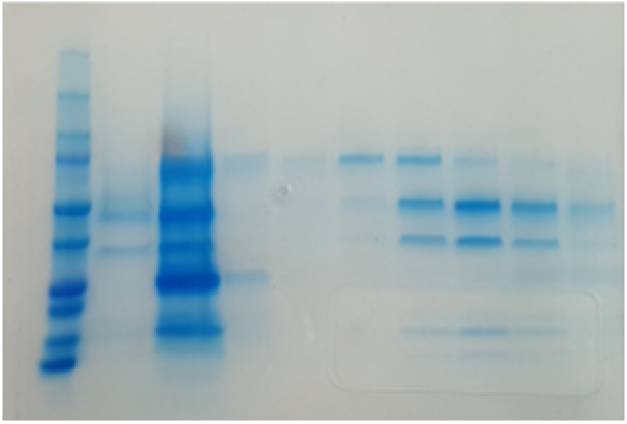

M = Bio-rad precission plus dual protein standards
FE = Flag elution
L = Sample loaded onto SEC
# = Volume sample was collected from SEC (ml)
